# Development and validation of the BD S_X_: a brief measure of mood and symptom variability for use with adults with bipolar disorder

**DOI:** 10.1186/s40345-016-0048-2

**Published:** 2016-03-01

**Authors:** Norm O’Rourke, Andrew Sixsmith, David B. King, Hamed Yaghoubi-Shahir, Sarah L. Canham, BADAS Study Team

**Affiliations:** Department of Public Health and Center for Multidisciplinary Research in Aging, Ben-Gurion University of the Negev, 8410501 Be’er Sheva, Israel; IRMACS Centre, Simon Fraser University, Burnaby, BC Canada; Gerontology Research Centre, Simon Fraser University, Vancouver, BC Canada; School of Computing Science, Simon Fraser University, Burnaby, BC Canada

**Keywords:** Bipolar disorder, Psychiatric status rating scales, Psychometrics, Reliability and validity

## Abstract

**Objectives:**

Ecological momentary sampling in BD research requires brief symptom measures with low cognitive demands to maximize data collection across the range of BD symptomatology. We developed the BD S_x_ cognizant of the challenges inherent in scale development with low prevalence populations and the limitations of existing measures (e.g., over-reliance on patients in acute states recruited from psychiatric settings). In order to be generalizable across the full spectrum of the illness, we also included those currently euthymic and those who avoid clinical contact.

**Methods:**

We recruited a global sample of 1010 adults with BD over 19 days using socio-demographically targeted, social media advertising and online data collection. At follow-up, 428 participants provided responses 67 days later on average. This enabled us to develop the BD S_x_ and replicate initial findings across multiple samples over time.

**Results:**

Both exploratory and confirmatory factor analyses support a 4-factor BD S_x_ model. Goodness of fit indices indicate good model fit across samples and over time. We labeled these factors: elation/loss of insight, affrontive symptoms of mania, cognitive/depressive, and somatic/depressive symptoms. Affrontive symptoms correlate positively with cognitive and somatic depression factors, which may suggest mixed-state symptom clusters in accord with DSM 5.

**Conclusions:**

Responses to the BD S_x_ reliably measure both depressive and hypo/manic symptoms. Temporal invariance analyses indicate that the 4-factor structure is consistent over time. Future research  should compare BD S_x_ responses to clinical diagnoses of hypo/mania and major depressive episodes.

## Background

Bipolar disorder (BD) is a chronic mental health condition defined by extremes of mood and mood variability (DSM-5 American Psychiatric Association 2013). Change in BD symptomatology can occur over the course of days, hours, and even minutes. As of yet, however, few if any self-report instruments exist to gauge change in symptom levels over short intervals. Existing scales were developed with non-clinical populations to measure normal mood, not symptomology [e.g., Profile of Mood States, POMS (Heuchert and McNair 2012); Positive and Negative Affect Scale, PANAS (Watson et al. 1998)].

A fundamental challenge in scale development with low prevalence populations such as BD is recruitment of samples of sufficient size given that psychometric research generally requires 200–300 participants (Clark and Watson 1995). As noted by Floyd and Widaman (1995), factor analysis conducted with small samples produces invalid or unstable factor structures unlikely to be replicated with other samples or generalize to the population. Moreover, scale development with clinical populations commonly relies on refractory patients or those in acute states in psychiatric settings (e.g., Michalak and Murray 2010). Yet research indicates that such samples are not representative of their respective populations (Dura and Kiecolt-Glaser 1990; Gallagher et al. 1989; Haberfellner 2000; Stirman et al. 2005). In other words, inpatient and outpatient BD samples commonly exclude those coping effectively, those supported by general practitioners in the community, and those who avoid clinical contact. However, valid and reliable test construction requires large samples recruited across multiple sites and settings (ideally multiple countries), reflecting the full range of BD symptomatology, including those who are currently euthymic. We developed the BD S_x_ cognizant of these challenges and the psychometric limitations of existing scales.

### Bipolar affective and older adults (BADAS) study

For our BADAS Study, we developed and validated BD S_x_ to enable ecological momentary sampling [EMS (Wenze and Miller 2010)] of hypo/manic and depressive symptoms using the iOS app we developed for iPad and iPhone (King et al. 2016). It is essential that EMS measurement be rapid and reliable with low *cognitive load* (Choi and Lee 2012). In order to minimize missing data, EMS questionnaires must be brief with low cognitive demands. This is especially true for BD research when participants are acutely symptomatic. For the BD S_x_, we adopted the format used for frequent measurement of mood and mood variability developed with non-clinical populations [e.g., POMS (Heuchert and McNair 2012), PANAS (Watson et al. 1998)]. In other words, we compiled a set of mood adjectives measuring both hypo/manic and depressive symptoms, asking respondents to specify how each describes how they feel right now, at this moment.

## Methods

### Participant recruitment

For the BADAS Study, we recruited 1010 adults with BD mostly from Canada, the US, UK, Ireland, South Africa, Australia, and New Zealand. This was achieved over 19 days using social media advertising targeted directly to those with BD. Participants were drawn from a global population of approximately 6.2 million English-speaking, adult Facebook users with ‘bipolar disorder interests’ (e.g., members of online BD support networks). Half the sample was 45+ years of age by design. As a response incentive, one randomly selected participant received a $500 lottery prize. The BADAS study was undertaken with full ethics approval from the Research Ethics Board at Simon Fraser University (2014s0375).

By clicking on advertisements appearing along the sidebar, or embedded within newsfeeds, prospective participants were directed to an online consent form that specified study inclusion criteria. Thereafter they completed a series of counterbalanced online questionnaires hosted on a secure university https server; responses were encrypted before transmission. The majority of participants provided an email address and consent to re-contact, allowing us to collect Time 2 responses 2 months later.

To corroborate that participants were in fact persons with BD, they were asked to specify their diagnosis (and BD subtype), date of birth, and country of residence at both points of measurement; rates of concurrence were 97, 96, and 97 %, respectively. In addition, participants were asked to list any prescribed psychotropic medications by category and comorbid conditions. Of those specifying medications, 95 % correctly listed and categorized both mood stabilizers and antidepressants, and 84 % for antipsychotics. Also, comorbid conditions and the relative frequency reported by participants correspond to epidemiological BD research [i.e., anxiety disorders most commonly cited (Lala and Sajatovic 2012)].

In light of participants’ ability to specify their prescribed medications with such accuracy (and concurrence between T1 and T2 responses to date of birth and country of residence questions), we contend it is very unlikely that participants invented or mispecified their BD diagnosis. And even if several concocted their identities, participating only to be included in the lottery,  our sample size was sufficiently large so that their responses would have had little or no impact on our findings (i.e., minimal measurement error). In no small degree, this is due to the fact that social media advertisements were directed specifically to prospective participants with BD and not other populations. This methodology has been used with a range of clinical and other circumscribed populations which we describe in detail elsewhere (King et al. 2014).

### Participant descriptive features

This sample was composed of 736 women, 266 men, and 6 specifying another gender (e.g., transgender). They ranged in age from 19 to 82 years, (mean) *M* = 45.28, (standard deviation) *SD* = 13.78. The largest proportion of participants lived in the U.S. (40 %) with 29 % in Canada, 17 % from the U.K., and 6 % from Ireland; smaller numbers living in Australia, New Zealand, and South Africa in descending order. In total, participants from 23 countries provided baseline responses; 90 % reported that they were Caucasian. Thirty per cent indicated that they were married whereas an almost equal number were currently single (28 %). A further 20 % were partnered (e.g., civil union) and 18 % separated or divorced. Only 2 % were widowed.

Of those reporting their specific diagnosis, roughly equal numbers indicated that they had been diagnosed with BD not otherwise specified (NOS 35 %) and BD II (34 %) whereas 27 % reported a BD I diagnosis. Eighteen per cent were unsure or unaware of their BD subtype. On average, participants had been diagnosed with BD 11.14 years ago (*SD* = 9.60, range 1 month–51 years), had 1.11 comorbid psychiatric conditions (*SD* = 1.38, range 0–6), and reported 2.47 prescribed medications (*SD* = 1.77, range 0–15). Most listed one or more mood stabilizer (58.9 %) and 1+ antidepressant (64.4 %) whereas smaller numbers listed 1+ anxiolytic (38.9 %) and 1 + antipsychotic (38.3 %). These percentages are commensurate with the BD subtypes reported by participants. By category, lithium (*n* = 78), bupropion (*n* = 43), clonazepam (*n* = 55), and quetiapine (*n* = 68) were the medications most commonly listed by participants (mood stabilizer, antidepressant, anxiolytic and antipsychotic, respectively). In their qualitative comments, several participants reported that they have discontinued pharmacotherapy (or now self-medicate with marijuana).

Although participants reported that they had completed 11.40 years of education on average (*SD* = 5.90, range 1–25), 21 % indicated that they were currently on sick leave or receiving a disability pension and 16 % were unemployed. Just 14 % were employed full-time, 13 % worked part-time, and 6 % were retired. Of those currently or previously employed, 25 % indicated that they worked in managerial/clerical position, 19 % were employed in professional/technical positions, 11 % in semi-skilled, and 10 % in skilled trades (e.g., electricians); the largest percentage (34 %) reported no paid work history. Consistent with previously published research (e.g., Hooshmand et al. 2014), these participants were largely under-employed relative to their education and qualifications.

### Instruments

*Patient Health Questionnaire (PHQ*-*9)* The PHQ-9 (Kroenke and Spitzer 2002) is a self-report version of the depression module from the PRIME-MD diagnostic interview (Spitzer et al. 1995). Participants respond to each of 10 PHQ-9 items (4 response options) based on problems they have experienced over the past 2 weeks. A total greater than 9 suggests significant symptomology with 89 % sensitivity and 88 % specificity vis-à-vis a diagnosis of a major depressive episode (Kroenke et al. 2001). The PHQ-9 has been used widely with unipolar and bipolar patient populations (Cerimele et al. 2014).

*Satisfaction with Life Scale (SLS)* The SLS (Diener et al. 1985) serves to measure perceived quality of life on the basis of person-specific criteria. Respondents compare their current circumstances against subjective standards to arrive at a global appraisal of life satisfaction (Diener 2000). Participants are presented with five separate questions with seven response alternatives (e.g., “In most ways my life is close to ideal”; “The conditions of my life are excellent”). Previously published research indicates that depression is associated with lower life satisfaction, whereas SLS is unrelated to hypo/manic symptoms of bipolar disorder (Meyer et al. 2004).

### Analytic strategy

Our 1010 participants were randomly assigned to one of three groups: 300 for initial exploratory factor analyses (EFA); 300 to a second EFA sample to replicate initial findings; and the remaining 411 participants were assigned to confirmatory factor analyses (CFA) to assess the measurement properties of the working version of the BD S_x_ derived on the basis of EFA findings. Participants responded to an initial pool of mood and symptom adjectives as well as the PHQ-9 and SLS. This was done in order to assess the concurrent and discriminant validity of BD S_x_ responses relative to established depression and satisfaction with life measures.

Sixty-seven days on average after initial recruitment, 428 participants again responded to the BD S_x_ along with other measures. We set out to replicate initial CFA findings; we then computed invariance analyses comparing T1 and T2 CFA models to assess the stability of BD S_x_ responses over time. This is a more appropriate psychometric procedure than computing test–retest reliability, as variability in mood is the hallmark of BD. Strong test–retest reliability would, in fact, indicate insensitivity to change over time. Temporal invariance analyses compare the underlying structure of scale responses irrespective of change in response levels. CFA models and invariance analyses were computed in accord with the procedures described by Byrne (2012).

## Results

### Study one

*Exploratory Factor Analyses (EFA)* We compiled a pool of 114 mood and symptom adjectives from existing scales, clinician input, and recommendations from our community advisory board. We then performed EFA with the first sub-sample of 300 participants. In accord with prior psychometric research with clinical populations (e.g., Chou and O’Rourke 2012), the likelihood method of factor extraction with varimax rotation was used. This initial pool of items demonstrated sufficient interrelatedness to undertake factor analysis according to the Kaiser–Meyer–Olkin (KMO) measure of sampling adequacy (KMO = .93).

The Kaiser-Guttman criterion initially suggested a 6-factor solution (i.e., eigenvalues >1); however, the Cattell-Nelson-Gorsuch (CNG) scree test indicated a 4-factor solution due to the notable leveling of eigenvalues after Factor 4. This 4-factor solution explained 49 % of observed variance. [As noted by Floyd and Widaman (1995), the Kaiser-Guttman criterion generally provides over-inclusive factor solutions.] Coefficient values for each of the top 10 items loaded on their respective factors with coefficient values greater than .30 as recommended (Tabachnick and Fidell 2013), most greater than .60.

EFA was next performed on a second subset of 300 participants with results very similar to the first EFA. A 4-factor solution was again most viable with the same items emerging as best measures of their respective factors, and in virtually the same sequence. Where two items had similar meaning, we selected the adjective suggesting symptomatology versus normal affect (e.g., hopelessness vs. sadness; hostile vs. angry; euphoric vs. happy). We provisionally selected 20 adjectives to measure these four BD S_x_ factors. Based on the meaning conveyed by each grouping, we labeled these factors: (1) cognitive/depressive; (2) somatic/depressive; (3) elation/loss of insight; and (4) affrontive symptoms of mania.

**Fig. 1:**
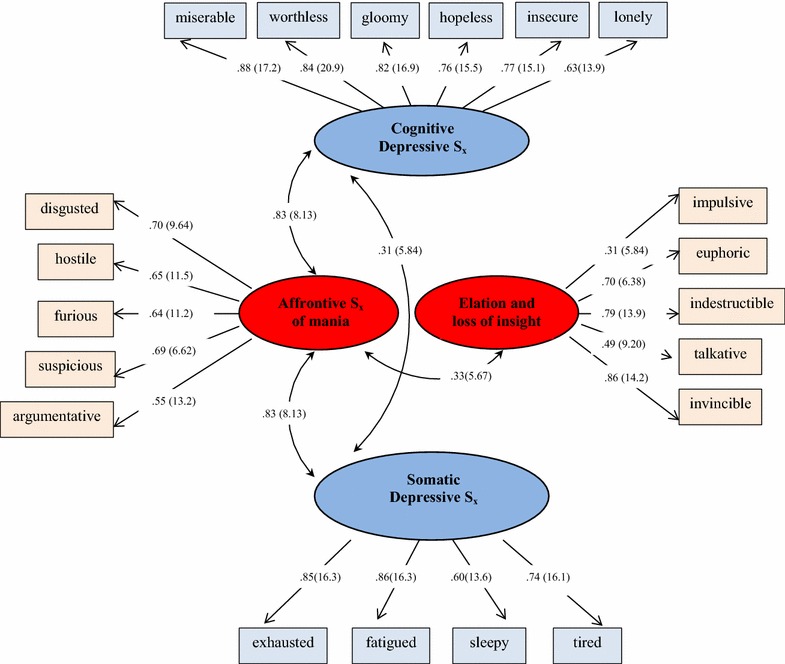
BD S_x_ 4-Factor Model of Symptomatology in Bipolar Disorder. Parameters expressed as maximum likelihood estimates (standardized solution). Parenthetical numbers indicate significance levels for parameter estimates (statistically significant *t* values >1.96)

*Confirmatory Factor analysis (CFA)* We next performed CFA to assess goodness of fit of this 4-factor model (*n* = 411). Each of the 20 items loaded significantly upon its respective factor (i.e., *t* values >1.96). Significant covariance was observed between the cognitive/depressive and somatic/depressive factors as well as the two hypo/mania factors (elation/loss of insight, affrontive symptoms). Also of note was significant covariance between the affrontive symptoms of mania and both cognitive and somatic depression factors suggesting clusters of mixed-state hypo/manic and depressive symptoms consistent with DSM 5 (2013). See Fig. 1.

Model fit was calculated subsequent to correcting for correlated error between 16 of 210 possible item pairs, *χ*^2^(df = 150) = 271.30, *p* < .01. Statistical power for this model was .99 (O’Rourke and Hatcher 2013). The Comparative Fit Index (CFI ≥ .95; CFI = .97), the Root Mean Square Error of Approximation (RMSEA ≤ .05; RMSEA = .045), and the full 90 % confidence interval for the RMSEA were each within ideal limits for this model [.036 < RMSEA CL_90_ < .053; see (Hu and Bentler 1999)]. The Standardized Root Mean Square Residual was in acceptable limits (SRMR ≤ .08; SRMR = .070). These results provide further support for the psychometric properties of the 4-factor model of BD S_x_ responses.

*Construct validity* The two BD S_x_ depression factors were strongly correlated (*r* = .55, *p* < .01) while the two hypo/mania factors were moderately correlated (*r* = .27, *p* < .01). We next computed correlation coefficients comparing responses to these four BD S_x_ factors with depressive symptoms and life satisfaction. As expected, both cognitive/depressive and somatic/depressive factors were strongly correlated with responses to the PHQ-9, *r* = .71, *p* < .01; *r* = .49, *p* < .01, respectively. Affrontive symptoms of mania were also correlated with participants’ responses to the PHQ-9 (*r* = .50, *p* < .01) whereas no association emerged for the elation/loss of insight factor, *r* = −.03, *p* = .38). These findings are consistent with CFA analyses showing significant covariance between affrontive symptoms of mania and both cognitive and somatic symptoms of depression, and no covariance between elation/loss of insight and either depression factor.

Responses to both the cognitive/depressive and somatic/depressive factors were significantly and inversely correlated with life satisfaction, *r* = −.42, *p* < .01; *r* = −.24, *p* < .01, respectively. Affrontive symptoms of hypo/mania were also negatively correlated with SLS, *r* = −.30, *p* < .01 whereas virtually no association was found between elation/loss of insight and life satisfaction, *r* = −.10, *p* = .05. These analyses provide support for the concurrent validity (cf. PHQ-9) and discriminant validity of responses to the BD S_x_ (cf. SLS). Both depression factors were strongly correlated with the PHQ-9 as well as the affrontive mania factor (but not the elation/loss of insight factor). Similarly, cognitive and somatic depression factors as well as affrontive symptoms of hypo/mania were inversely associated with life satisfaction. Consistent with previous research (e.g., Meyer et al. 2004), elation/loss of insight was unrelated to life satisfaction.

### Study two

Of those providing Time 1 data, 97 % (or 917 of 1011) provided us with an email address and permission to contact them in future. And 47 % (or 428 of 917) responded to our request for Time 2 responses roughly 2-months after initial data collection. This rate of retention is high for questionnaire research (DeVellis 2012).

An equal proportion of men and women responded at both T1 and T2 versus T1 only, *χ*^2^(df = 2) = .09, *p* = .96. The ethnic composition of groups was similar [*χ*^2^(df = 8) = 9.50, *p* = .30]; they were roughly the same age [*M* = 45.28 years, range 19–82; t(995) = .97, *p* = .33] and the relationship status of participants in both groups was comparable, *χ*^2^(df = 7) = .09, *p* = .13. Moreover, those who responded at both points of measurement reported the same numbers of comorbid conditions, *t*(1008) = .73, *p* = .50 and medication adherence, t(341) = .64, *p* = .52. Yet those providing only T1 responses were less educated (*n* = 11.02 years) than those providing both T1 and T2 responses [*n* = 12.15; *t(1001)* = 2.88, *p* < .01] and were of lower socioeconomic status based on work performed now or prior to retirement *χ*^2^(df = 6) = 16.78, *p* = .01. Yet those lost to follow up were largely indistinguishable from the full T1 sample. In other words, attrition between points of data collection does not appear to be a confounding factor.

We computed a second CFA to assess the 4-factor BD S_x_ model, and once again, each item contributed significantly to measurement of its respective factor (i.e., *t* values >1.96). Statistical power for this CFA model was again calculated as .99 (O’Rourke and Hatcher 2013). Goodness of fit indices were within acceptable to ideal parameters, *χ*^2^(df = 160) = 237.91, *p* < .01. More precisely, the CFI = .97, the RMSEA = .042, and the full 90 % confidence interval for the RMSEA was within ideal parameters (.030 < RMSEA CL_90_ < .053); the SRMR (.066) was in acceptable limits. These results replicate initial CFA findings and provide further confirmation of psychometric soundness of the BD S_x_.

*Temporal Analyses.* Invariance analyses were next performed to test the temporal stability of the 4-factor model and the measurement reliability of BD S_x_ items over time. CFA models, initially run separately, were next computed simultaneously. Covariance estimates and path coefficients for this 4-factor BD S_x_ model were anchored in sequence to ascertain if and where significant differences might exist between T1 and T2 CFA models (i.e., significant change in the chi-square statistic). See Table 1.

**Table 1:** Summary specifications and temporal invariance analyses comparing Time 1 and Time 2 BD S_x_ responses

Model	χ^2^	df	Δχ^2^	Δdf	SRMR	CFI	RMSEA (CL_90_)
Baseline model	518.35	307	–	–	.0701	.97	.030 (.026–.035)
Cognitive–somatic	518.40	308	.05	1	.0699	.97	.030 (.026–.035)
Affront–elation/insight	518.43	309	.03	1	.0700	.97	.030 (.026–.035)
Affront–cognitive	518.84	310	.39	1	.0696	.97	.030 (.026–.035)
Affront–somatic	518.84	311	.05	1	.0697	.97	.030 (.026–.034)
Cognitive/depressive	529.54	316	10.65	5	.0698	.97	.030 (.026–.035)
miserable	522.23	312	3.34	1	.0697	.97	.030 (.026–.035)
worthless	523.30	313	.02	1	.0697	.97	.030 (.026–.035)
gloomy	523.20	314	.01	1	.0697	.97	.030 (.026–.034)
hopeless	525.21	315	2.22	1	.0697	.97	.030 (.026–.034)
insecure	529.42	316	.20	1	.0698	.97	.030 (.026–.035)
lonely	529.54	316	.12	1	.0696	.97	.030 (.026–.035)
Somatic/depressive	530.07	319	.53	3	.0697	.97	.030 (.026–.035)
fatigued	529.89	317	.35	1	.0696	.97	.030 (.026–.035)
sleepy	530.01	318	.20	1	.0697	.97	.030 (.025–.034)
exhausted	530.07	319	.06	1	.0697	.97	.030 (.025–.034)
tired	530.07	319	.16	1	.0697	.97	.030 (.025–.034)
Elation/loss of insight	538.24	323	8.17	4	.0692	.97	.030 (.026–.035)
impulsive	535.53	320	5.46*	1	.0692	.97	.030 (.026–.035)
indestructible	535.54	321	.01	1	.0692	.97	.030 (.026–.035)
talkative	535.83	322	.29	1	.0694	.97	.030 (.026–.035)
invincible	538.24	323	2.41	1	.0691	.97	.030 (.026–.035)
euphoric	538.24	323	.29	1	.0689	.97	.030 (.026–.035)
Affrontive S_x_ of mania	543.93	327	5.69	4	.0686	.97	.030 (.026–.035)
hostile	542.81	324	4.57*	1	.0686	.97	.030 (.026–.035)
furious	543.15	325	.34	1	.0686	.97	.030 (.026–.034)
argumentative	543.79	326	.64	1	.0686	.97	.030 (.026–.034)
disgusted	543.93	327	.14	1	.0686	.97	.030 (.026–.034)
suspicious	543.93	327	.80	1	.0686	.97	.030 (.026–.034)

* p < .05; ** p < .01

*df* degrees of freedom, *SRMR* Standardized Root Mean Square Residual, *CFI* Comparative Fit Index; *RMSEA* Root Mean Square Error of Approximation, *RMSEA* *CL*_*90*_ 90 % Confidence Limits for the RMSEA statistic

Covariance between the four BD S_x_ factors did not differ between points of measurement supporting the factorial validity of the latent structure of BD S_x_ responses over time. Also of note, the relative strength of association between items and their respective factors differed for 2 of 20 items (i.e., hostile, impulsive) yet these differences are negated by overall measurement consistency of these items’ respective factors. In other words, responses to these BD S_x_ items are highly reliable and temporal analyses demonstrate considerable consistency of measurement over time.

Time 2 data allowed us to replicate the 4-factor model; moreover, this factor structure appears valid and items appear reliable over time. These findings provide further confirmation of the psychometric properties of responses to this scale. We present the BD S_x_ in Table 2 for use in EMS research and other studies that require rapid, valid and reliable measurement of both hypo/manic and depressive symptoms. The BD S_x_ can also be used in clinical practice to assess change over the course of treatment, and in randomized controlled studies when BD symptoms are measured repeatedly.

**Table 2:** BD S_x_: list of words that describe feelings people have. After reading each, please indicate how the word reflects how you feel—*right now, at this moment*: *0* not at all, *1* a little, *2* a lot

1.	miserable	0	1	2
2.	talkative	0	1	2
3.	tired	0	1	2
4.	hostile	0	1	2
5.	disgusted	0	1	2
6.	impulsive	0	1	2
7.	worthless	0	1	2
8.	furious	0	1	2
9.	sleepy	0	1	2
10.	indestructible	0	1	2
11.	insecure	0	1	2
12.	fatigued	0	1	2
13.	gloomy	0	1	2
14.	invincible	0	1	2
15.	exhausted	0	1	2
16.	euphoric	0	1	2
17.	lonely	0	1	2
18.	suspicious	0	1	2
19.	hopeless	0	1	2
20.	argumentative	0	1	2

*Scoring key:*

Cognitive symptoms = 1, 7, 11, 13, 17, 19

Somatic symptoms = 3, 9, 12, 15

Affrontive symptoms = 4, 5, 8, 18, 20

Elation/loss of insight = 2, 6, 10, 14, 16

## Discussion

The results of this study provide considerable psychometric support for the reliability and validity of responses to the BD S_x_. A working version of this scale was first developed on the basis of separate EFA analyses later supported by CFA, each using large samples. At follow-up, we replicated this 4-factor model and demonstrated strong temporal consistency across two points of measurement (*n* = 428). This level of methodological rigor distinguishes development of the BD S_x_ from prior scales developed for BD research and clinical practice.

We also compared the BD S_x_ to established measures of life satisfaction and depressive symptomatology. Responses to cognitive/depressive and somatic/depressive factors, and affrontive symptoms of mania, were each positively and significantly correlated; elation/loss of insight was unrelated to either cognitive or somatic symptoms of depression. Similarly, the elation/loss of insight was uncorrelated with life satisfaction whereas the remaining three factors are inversely related to satisfaction with life. These analyses demonstrate the concurrent and discriminant validity of responses to the BD S_x_.

It might seem counterintuitive that elation/loss of insight is not positively correlated with life satisfaction, though as we note, this finding is consistent with previously published research (Meyer et al. 2004). This may be because life satisfaction is a cumulative, global appraisal of one’s life to this point (Diener 2000) in contrast to momentary mood at one point. Future research should compare BD S_x_ responses to hypo/mania self-report measures to further support the psychometric properties of this scale.

Of further note is the significant correlation between affrontive symptoms of hypo/mania and both depression factors. No such correlation exists between elation/loss of insight and neither cognitive nor somatic depression factors, as we would anticipate. The former finding is consistent with evolving understanding of mixed-symptom BD symptomatology (DSM-5 American Psychiatric Association 2013) as manic and depressive symptoms are not inversely associated but can present concurrently for many or most with BD. This is in accord with the 4-factor model of BD S_x_ responses in which depression and hypo/mania are measured as distinct but correlated constructs.

Our findings suggest that both symptoms of depression and mania fall into two separate groupings. Consistent with unipolar depression research (Carney and Freedland 2012), depressive symptoms appear to cluster in distinct somatic and cognitive groupings. Maybe more noteworthy is the composition of hypo/mania factors. The elation/loss of insight factor appears to capture mania as traditionally envisioned (e.g., elation, expansive mood) along with disconnect from reality (e.g., invincibility, indestructibility). In contrast, the affrontive symptoms capture the irritable and ill-tempered ways in which mania can present, especially with older adults (Krauss Whitbourne 2000). This factor is positively correlated with each of the other three, including both depressive factors—and more strongly with cognitive and somatic factors of depression than elation/loss of insight.

The large, international sample of respondents recruited for this study is a definitive strength; follow-up data collection confirmed initial findings and demonstrated measurement consistency over time. These findings support both the validity and reliability of the BD S_x_ over time. Although participants were drawn from all English-speaking countries, comparatively few persons with BD from minority communities were recruited. Further study is warranted with more representative samples and research is needed comparing BD S_x_ responses relative to clinical assessment of depression and hypo/manic mood episodes.

### Ongoing BD research

For the BADAS Study, we have developed both am (morning) and pm (afternoon/evening) versions of our iOS app. Participants complete the BD S_x_ as well as sleep quality and medication adherence (am version) and any important events of the day (pm version). At enrolment, participants specify windows of general availability and, by corollary, times of day when generally unavailable. They are randomly prompted twice daily within 30-minute windows and receive up to three prompts (initial, 20, and 25 min thereafter). If they respond after the first (or second) prompt, they do not hear the second (or third) prompt. Participants can also submit voluntary questionnaires at any point if something significant occurs that they wish to report.

EMS responses are time- and GPS-stamped so that we can determine if skipped questionnaires are missing at random or missing systematically (e.g., while manic only). Recent research indicates that smartphone sensors can predict BD symptomatology with considerable accuracy (Osmani et al. 2015). By measuring hypo/manic and depressive symptoms and symptom variability with the BD S_x_, we can include this subjective information to further refine person-specific algorithms to better identify predictors of mood episodes and factors associated with wellness with BD over time. Data collection for the BADAS Study is underway.
